# Quantifying triglyceride-rich lipoprotein atherogenicity, associations with inflammation, and implications for risk assessment using non-HDL cholesterol

**DOI:** 10.1016/j.jacc.2024.07.034

**Published:** 2024-10-01

**Authors:** Elias Björnson, Martin Adiels, Anders Gummesson, Marja-Riitta Taskinen, Stephen Burgess, Chris J Packard, Jan Borén

**Affiliations:** 1 Department of Molecular and Clinical Medicine, University of Gothenburg, Gothenburg, Sweden; 2 School of Public Health and Community Medicine, Institute of Medicine, University of Gothenburg, Gothenburg, Sweden; 3 Region Västra Götaland, Sahlgrenska University Hospital, Department of Clinical Genetics, Gothenburg, Sweden; 4 Research Program for Clinical and Molecular Metabolism, University of Helsinki, Helsinki, Finland; 5 MRC Biostatistics Unit, University of Cambridge, Cambridge, UK; 6 Cardiovascular Epidemiology Unit, Department of Public Health and Primary Care, University of Cambridge, Cambridge, UK; 7 Institute of Cardiovascular and Medical Sciences, University of Glasgow, Glasgow, UK

**Keywords:** Mendelian randomisation, cardiovascular disease, TRLs, LDL, UK Biobank

## Abstract

**Background:**

Triglyceride-rich lipoproteins and remnants (TRL/remnants) have a causal but not yet quantified relationship with coronary heart disease (CHD - myocardial infarction plus revascularisation).

**Objectives:**

To estimate TRL/remnant per-particle atherogenicity, investigate causal relationships with inflammation, and determine whether difference in the atherogenicity of TRL/remnants and LDL impact the causal association of non-HDL-C with CHD.

**Methods:**

Single nucleotide polymorphisms (SNPs) (n=1357) identified by genome-wide association in the UK Biobank were ranked into 10 clusters according to the effect on TRL/remnant-C versus LDL-C. Mendelian randomisation analysis was used to estimate for each SNP cluster CHD odds ratios per 10 mg/dL apoB and per 0.33 mmol/L non-HDL-cholesterol, and to evaluate association of TRL/remnants with biomarkers of systemic inflammation.

**Results:**

SNPs in cluster 1 predominantly affected LDL-C while SNPs in cluster 10 predominantly affected TRL/remnant-C. CHD risk per genetically predicted increase in apoB and in non-HDL-C rose across clusters. Odds ratio per 10 mg/dL higher apoB was 1.15[95%CI:1.11-1.19] in cluster 1 versus 1.70[95%CI:1.52-1.90] in cluster 10. Comparing odds ratios between these TRL/remnant predominant- and LDL predominant-clusters, we estimated that TRL/remnants were at least 3.9[95%CI:2.8-5.4] times more atherogenic than LDL on a per-particle basis. For non-HDL-C, CHD odds ratios per 0.33 mmol/L rose from 1.15[1.11−1.19] for cluster 1 to 1.40[1.30−1.50] for cluster 10. TRL/remnants exhibited causal relationships with inflammation, but this did not explain their greater atherogenicity.

**Conclusion:**

TRL/remnants are about 4 times more atherogenic than LDL. Variation in the causal association of non-HDL-C with CHD indicates that adjustment for percentage TRL/remnant-C may be needed for accurate risk prediction.

## Abbreviations

apoB: apolipoprotein B
CHD: coronary heart disease
GWAS: genome-wide association studies
LDL-C: low density lipoprotein cholesterol
MR: Mendelian randomisation
Non-HDL-C: non-high density lipoprotein cholesterol
SNPs: single nucleotide polymorphisms
TRLs: triglyceride-rich lipoproteins
TRL/remnant-C: triglyceride-rich lipoproteins/remnant cholesterol

## Introduction

Triglyceride-rich lipoproteins (TRL) have been implicated as causal agents in atherogenesis. The strongest evidence for this comes from studies in which variants in genes known to alter circulating triglyceride (TG) and hence TRL concentrations are associated with coronary heart disease (CHD) outcomes.^1–5^ The availability of large cohorts with extensive genotype and phenotype data has enabled exploration of the relative atherogenicity of TRL and their remnant particles (TRL/remnants) versus LDL on a per-particle basis (i.e., per unit apoB since TRL and LDL particles each contain one apoB protein).^2–6^ Contrary to earlier reports,^3–5^ it was demonstrated recently that TRL/remnants appeared to have a stronger association with CHD risk than LDL.^2^ One explanation for this might be lie in the higher cholesterol content of TRL/remnants compared to LDL.^1^ Another possibility arises from the fact that atherosclerosis has an inflammatory component, and there is evidence of a strong association between plasma TG and biomarkers of chronic inflammation.^7–10^ However, the extent to which the relationship between TRL/remnants and chronic inflammation explains the causal association of these particles with CHD risk remains unclear.

In our previous study we identified two clusters of SNPs that had differing effects on TRL/remnants versus LDL; the SNP cluster that gave higher genetically predicted TRL/remnant levels was associated with a higher CHD risk per unit increase in apoB.^2^ The present investigation extended this SNP cluster-based approach to provide a quantitative estimate of the relative atherogenicity of TRL/remnant particles. Further, we explored the hypotheses that (a) a causal association of TRL/remnants with inflammation may help explain their greater atherogenicity, and (b) the difference in the strength of association of TRL/remnants and LDL with risk implies that the quantitative relationship of non-HDL cholesterol - the sum of TRL/remnant-C and LDL-C – to CHD risk is variable: a clinically important issue since this is a widely used index for risk assessment and treatment decisions.

## Methods

### Study population

The cohort comprised the UK Biobank with over 502,000 UK residents of predominantly white ancestry, who had the required biochemical and genetic data available.^11^ Genetic instruments were derived from subjects who were not on lipid-lowering therapy at baseline (see **Online Figure 1)**. All subjects including those on lipid-lowering therapy were used to assess the association of genetically predicted lipoprotein variables with CHD risk.

### Lipid and lipoprotein measurements

LDL-C was measured directly (Beckman Coulter, Brea, CA). Non-HDL cholesterol was calculated as the difference between plasma cholesterol and HDL cholesterol.^11,12^ TRL/remnant-C was derived by subtracting direct LDL-C from non-HDL-C.^1^ Other analytes were measured by standard laboratory methods. Blood samples were not obtained in the fasting state, so postprandial lipoproteins were possibly present.

### Inflammatory biomarkers

C-reactive protein (CRP), white blood cell count, and blood neutrophil count were measured in the UK Biobank using standard methods. Glycoprotein acetyls, a stable inflammatory biomarker,^13,14^ was determined by NMR.

### Genetic analyses

Genotyping with the UK BiLEVE Axiom or UK Biobank Axiom arrays provided an evaluation of 805,426 single nucleotide polymorphisms (SNPs) spanning the entire genome (**Online Figure 1)**.

### Genome-wide association study

New GWAS adjusted for age, sex, BMI and genetic principal components 1-5 were performed to identify SNPs associated with plasma TG, TRL/remnant-C or LDL-C. SNPs meeting the significance threshold of *p*<5×10^-8^ were pruned for linkage disequilibrium (r^2^ <0.1 with a window size of 20Mb) and minor allele frequency (threshold >0.01). If two SNPs were in linkage disequilibrium, the SNP with the largest combined effect size (square root of [LDL-C effect size squared plus TRL/remnant-C effect size squared]) was selected. The list was further filtered for association (Bonferroni-Holms adjusted P<0.05) with lipoprotein(a) which excluded 28 SNPs. This process resulted in a final set of 1,357 SNPs (**Online Figure 1**).

### Definition of SNP clusters

SNPs identified by GWAS were ranked according to the ratio of their effect sizes (β-coefficients) for TRL/remnant-C relative to LDL-C. The ranked SNPs were then divided into 10 clusters (decile analysis) or 20 clusters (ventile analysis) with each cluster having an equal number of SNPs. (**Online Figure 1)**.

### CHD outcomes

These are defined in **Online Table 1**. For studies of the association of CHD with genetically predicted lipid levels, outcomes were the combination of prevalent and incident CHD events (myocardial infarction (MI) and coronary revascularisation).

### Statistical methods

Statistical analyses were performed using R version 4.0.4. Multivariable Mendelian randomisation (MR) analyses based on the inverse variance-weighted method (which assumes that all variants are ‘valid’ instrumental variables; that is the SNP effect on CHD outcome is solely through its effect on the exposure/risk factor^15,16^), were performed to determine genetic associations between lipoprotein variables, and causal associations of these variables with CHD outcomes. β-coefficients were derived using exposure data from subjects who had all required lipid measurements and were not receiving lipid-lowering treatment. Associations with CHD outcomes were determined using data from all subjects (including those on treatment) with available lipid measurements. Note that there was no requirement to adjust multivariable Mendelian randomisation models for risk factors such as age, sex, diabetic status, HDL-cholesterol etc. since these models are genetic based and not confounded by lifestyle or other covariates.

For each SNP cluster two odds ratios for CHD were calculated, one per standardised increase in apoB (10 mg/dL) and the other per standardised increase in non-HDL-C (0.33 mmol/L equal to 13 mg/dL). This degree of non-HDL-C exposure was chosen so to scale odds ratios for non-HDL-C to be of the same order of magnitude as those for apoB. These two odds ratios were then related to percentage TRL/remnant-C in non-HDL-C (that is the genetically determined effect size on TRL/remnant-C divided by the genetically determined effect size on non-HDL-C for each SNP averaged over all the SNPs in the cluster). The rationale underlying calculation of per-particle atherogenicity of TRL/remnants versus LDL using SNP clusters is explained in detail in **Online Figure 2**.

Mediation analysis was performed for specific SNP clusters with the largest effect size for TRL/remnants using the R-package *mediation* and using a multivariable Mendelian randomisation approach. The objective was to test whether the causal association of apoB (present predominantly in TRL/remnants in the SNP clusters tested) with CHD was mediated by a causal effect on inflammatory processes as assessed by biomarkers of chronic systemic inflammation.

## Results

The UK Biobank cohort comprises 502,460 men and women with a mean age of 56.5 years at enrolment. GWAS were conducted to identify SNPs related to TG, TRL/remnant-C or LDL-C in subjects not on lipid-lowering medication at baseline. The numbers of individuals with these lipoprotein levels available was 383,983 for TG, 350,797 for TRL/remnant-C and 383,566 for LDL-C. Mendelian randomisation analyses were conducted in 488,171 subjects both on and off lipid-lowering medication. Numbers of CHD (MI or revascularisation) events for prevalent (baseline) and incident outcomes are given in **Online Table 1**.

### Mendelian randomisation models of CHD risk incorporating apoB and lipid variables

A total of 1,357 SNPs associated with TG, TRL/remnant-C or LDL-C were identified using GWAS. The SNPs exhibited a wide range of effect sizes for these lipid variables relative to each other as shown in Online Figure 3. In multivariable Mendelian randomisation analyses using this expanded SNP set (compared to that reported earlier),^2^ we confirmed that TRL/remnant-C and TG remained independently associated with CHD risk as causative factors when apoB was included in the model (Table 1 **Models 1 and 2**). Further, in a model containing non-HDL-C and apoB, the former was found to be the sole significant causative factor; apoB became non-significant with a odds ratio of 1.0 (Table 1 **Model 3**). Comparing TRL/remnant-C and LDL-C (per 0.33 mmol/L increase) directly indicated that the cholesterol present in TRL/remnant particles was associated with a higher genetically predicted risk than that carried in LDL particles (Table 1 **Model 4**).

These findings raise questions concerning the magnitude and physicochemical basis of the higher per particle atherogenicity for TRL/remnants relative to LDL which we address below using a SNP cluster-based approach similar to that described previously.^2^

### Association of genetically predicted variation in apoB with risk of CHD by SNP cluster

**Figure 1:**
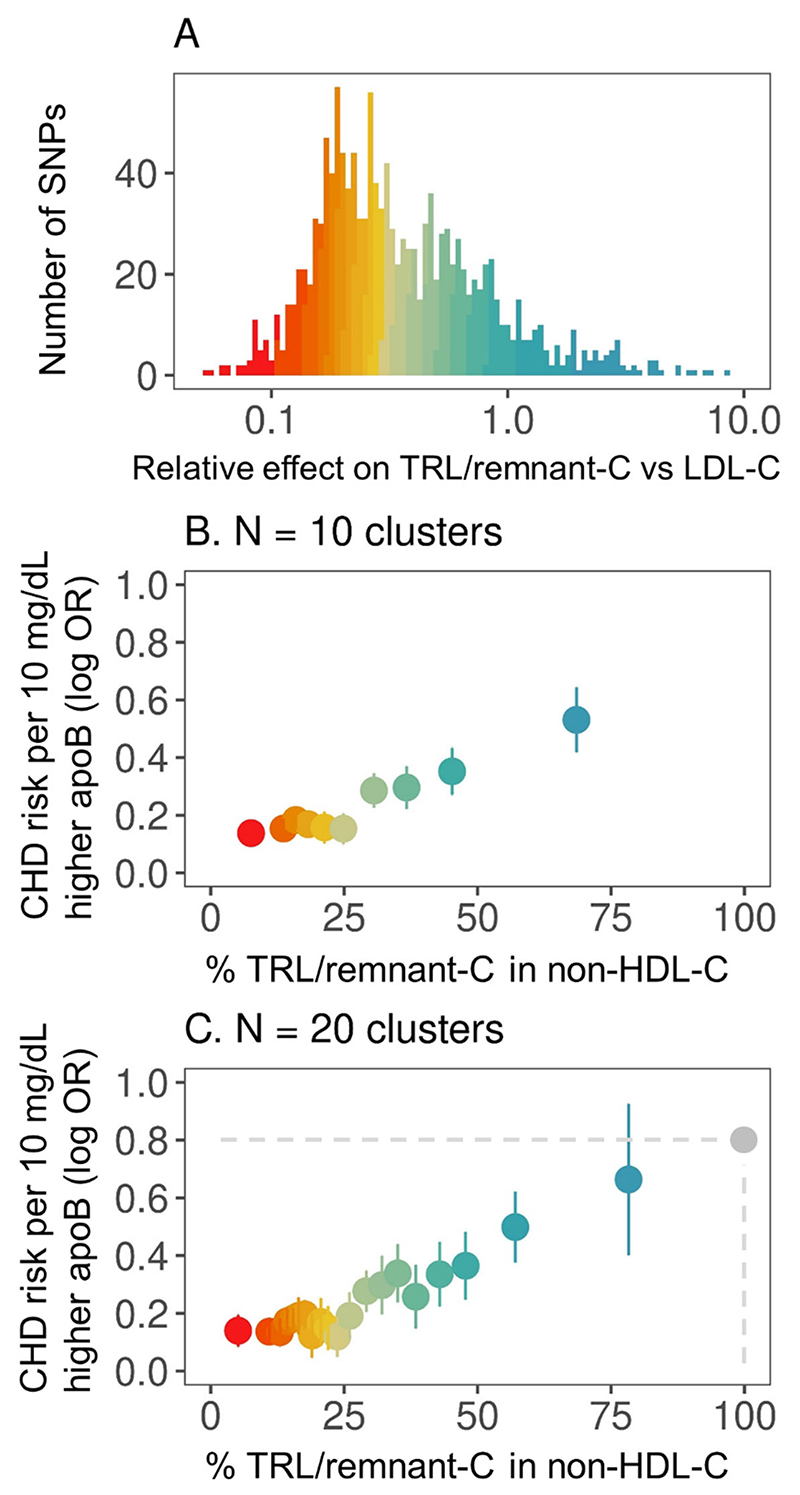
CHD risk per standardised change in apoB across SNP clusters. **Panel A:** SNPs (n=1357) were ranked according to the ratio of effect sizes (β-coefficients) for TRL/remnant-C and LDL-C. The SNPs were then grouped into clusters of equal size, 10 clusters for the decile analysis in **Panel B**, 20 clusters for the ventile analysis in **Panel C**. **Panel B:** For each of the 10 SNP clusters, the mean %TRL/remnant-C in non-HDL-C was calculated (effect size for TRL/remnant-C divided by the effect size for non-HDL-C and an average percentage taken over all the SNPs in a cluster). Mendelian randomisation analysis was used to estimate for each of the 10 SNP clusters CHD risk per 10 mg/dL increase in apoB (expressed as odds ratios (OR) and standard errors). Note that for each of the 10 SNP clusters effect sizes were determined across the whole UK Biobank cohort (that is it was the SNPs in Panel A that were clustered into deciles not the population on which they were tested.) **Panel C:** The SNP set was divided into 20 clusters (ventiles) to expand the range of genetically determined variation in TRL/remnant-C. Analyses then proceeded as described for Panel B. Extrapolation by regression to a theoretical 100%TRL/remnant-C in non-HDL-C yielded the odds ratio shown by the grey circle.

To derive sets of SNPs that differed markedly in their effects on TRL/remnants versus LDL, all 1,357 SNPs were ranked according to their effect size (β-coefficient) for TRL/remnant-C relative to their effect size for LDL-C (Figure 1 **Panel A**). The entire SNP set was then divided into 10 clusters (deciles) which had genetically predicted TRL/remnant-C to LDL-C ratios that varied more than 15-fold, from <0.2 in SNP cluster 1 to >3 in SNP cluster 10.

We then calculated for each SNP cluster the average effect of the included SNPs on TRL/remnant abundance using the entire cohort of subjects. That is, for each SNP the effect size on TRL/remnant-C was determined as a percentage of the effect size on non-HDL-C, and an average taken over all SNPs in the cluster (Table 2, Figure 1 **Panel B**). As shown in Table 1, for the 136 SNPs in cluster 1 the average TRL/remnant-C effect size across all subjects was 7.6% of the non-HDL-C effect size with 92.4% of non-HDL-C being attributed to the effect on LDL-C. Conversely, for the 135 SNPs in cluster 10 the average effect size across all subjects for TRL/remnant-C was 68.5% of non-HDL-C, and 31.5% was LDL-C (Table 2).

Next, Mendelian randomisation analyses were performed to determine for each SNP cluster the CHD risk per standardised increase (10 mg/dL) in apoB. As shown in Figure 1 **Panel B** and Table 2, across the 10 SNP clusters there appeared to be a positive, graded relationship between genetically predicted %TRL/remnant-C in non-HDL-C and the CHD risk per 10 mg/dL increase in apoB; for SNP cluster 1 the odds ratio was 1.15 [95% CI: 1.11−1.19] while for SNP cluster 10 the odds ratio was substantially and significantly higher at 1.70 [95% CI: 1.52−1.90] (see also **Online Table 3** which shows that further MR analyses using methods tolerant of potential SNP pleiotropic effects provide similar results to Table 1) (Central Illustration).

A similar pattern was observed when the CARDIoGRAMplusC4D cohort was used in a replication analysis. A total of 1,264 of the 1,357 SNPs were present in this data set and the SNP cluster structure was well represented. As can be seen in **Online Figure 4**, the results were in close agreement with those in Figure 1B.

In sensitivity analyses, we tested if the pattern observed in Figure 1B differed if MI alone was used as the outcome. Results were virtually identical to those with the combined endpoint (**Online Figure 5**). The number of revascularisations was too small to use this outcome alone. A further concern was that low frequency abnormal phenotypes that affect TRL/remnant abundance may exert a disproportionate influence over the relationship seen in Figure 1**B**. The most likely candidate is apoE2 homozygosity which causes a dysbetalipoproteinaemia characterised by increased remnant levels. Repeating the analysis excluding subjects homozygous for apoE2 (rs7412) had no discernible effect on the relationship (**Online Figure 5**).

### Application of SNP cluster approach to an exploration of TRL/remnant atherogenicity

Definition of the gradient between %TRL/remnant-C in non-HDL-C and CHD risk per 10mg/dL total apoB in Figure 1B allowed us to explore the following key questions:

- Can a quantitative estimate be derived for the atherogenicity (CHD risk per particle) of TRL/remnants relative to LDL?
- Can the higher per-particle atherogenicity of TRL/remnants be attributed to a causal relationship with chronic inflammation?
- Is the CHD risk per standardised change in non-HDL-C constant or variable since its components (TRL/remnant-C and LDL-C) exhibit differing strengths of causal association with CHD?

### Per-particle atherogenicity of TRL/remnants relative to LDL

As set out in the rationale in **Online Figure 2**, to generate a quantitative estimate of the atherogenicity of TRL/remnants relative to LDL the SNP effects in Figure 1 **Panel B** would ideally have spanned the entire range, that is where genetically predicted variation in non-HDL-C was due 100% to LDL-C at one end and due 100% to TRL/remnant-C at the other. For the SNP clusters at the extremes of the range, we could then have assigned the 10mg/dL change in total apoB entirely to LDL and TRL/remnant particles respectively and compared the CHD odds ratios to calculate a relative per-particle atherogenicity.

However, in the decile analysis (Figure 1B) while SNP cluster 1 yielded an average effect size for non-HDL-C that was >92% due to LDL-C, the average effect size for TRL/remnant-C in SNP cluster 10 was only 68.5% of that for non-HDL-C. Thus, the value (with uncertainty limits) obtained by dividing the CHD (log) odds ratio per 10 mg/dL apoB for cluster 10 by the corresponding value for cluster 1 (Table 3) at 3.9 [95%CI: 2.8−5.4] is a minimum/ conservative estimate for the relative atherogenicity of TRL/remnant versus LDL particles. A replication analysis undertaken using the CARDIoGRAMplusC4D cohort (with MI as outcome) gave a value of 2.9 [95%CI: 1.8−4.3] for relative atherogenicity (again comparing decile 10 with decile 1) (Table 3, **Online Figure 4**), a figure in broad agreement (confidence limits overlapped) with the estimate obtained from the UK Biobank.

To expand the range of genetically determined variation in %TRL/remnant abundance we divided the 1357 UK Biobank SNP set into 20 clusters (ventiles) (Figure 1 **Panel C**). Now, for SNP cluster 1 genetic variation in non-HDL-C was about 95% due to LDL-C while in SNP cluster 20 80% of variation in non-HDL-C was due to TRL/remnant-C. This ventile analysis extended the experimental observations towards the upper end of the range (at the expense of wider confidence limits) and allowed us to allocate the 10mg/dL change in plasma apoB in cluster 20 to a predominant increase in TRL/remnant particle number (Figure 1C, **Online Figure 2**). Comparing the CHD risk (log) odds ratios per 10 mg/dL increase in apoB in cluster 20 versus cluster 1 gave the higher (but still conservative) value of 4.7 [95%CI: 2.6−8.6] for the relative atherogenicity of TRL/remnants versus LDL.

In a further exploratory analysis, the gradient in Figure 1C was extrapolated (using regression) to the point where 100% of the genetically determined change in non-HDL-C was in theory due to TRL/remnant-C. This was associated with a projected log odds ratio per 10 mg/dL apoB of 0.8. Attributing this CHD risk to an increase solely in TRL/remnant particles and comparing the log odds ratio to that observed for LDL in cluster 1 of the ventile analysis gave a per-particle relative atherogenicity (0.8 divided by 0.14) of 5.8.

### Association of TRL/remnants with biomarkers of systemic inflammation

**Figure 2:**
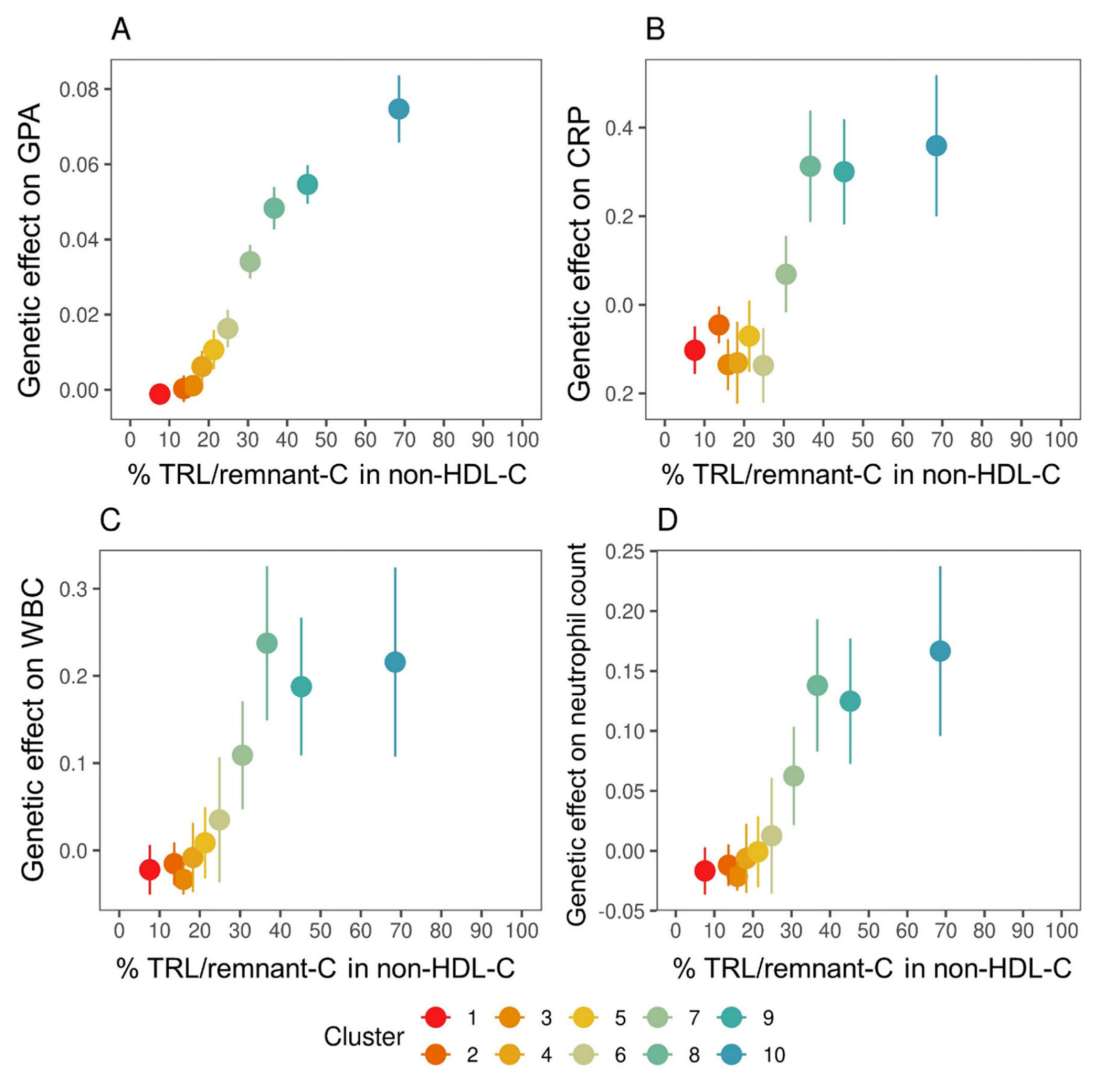
Association of genetically predicted TRL/remnant-C with inflammation biomarkers. Genetically determined changes in glycoprotein acetyls (GPA), C-reactive protein (CRP), white blood cell count (WBC), and neutrophil count per 10 mg/dL increase in apoB were related to genetically predicted percentage TRL/remnant-C in non-HDL-C determined for each SNP cluster in the decile analysis shown in Figure 1 **Panel B**.

For each of the 10 SNP clusters in Figure 1A, Mendelian randomisation analysis was used to relate %TRL/remnant-C in non-HDL-C to glycoprotein acetyls, C-reactive protein (CRP), white blood cell count, and blood neutrophil count (Figure 2). To allow comparison across clusters the genetically predicted change in inflammatory biomarkers was standardised per 10 mg/dL apoB. In clusters 1 to 4 where SNPs had effects mainly on LDL-C there was minimal impact on these inflammatory markers, but for SNP clusters 5 to 10 as the %TRL/remnant-C in non-HDL-C rose so did the causal effect size for all four biomarkers per 10 mg/dl apoB (per particle) (Figure 2).

Next, we conducted a mediation analysis to ascertain if variation in the inflammatory biomarkers explained statistically the association of TRL/remnant-C with CHD risk. Both the *mediation* package and multivariable Mendelian randomisation analyses were undertaken for SNP clusters 9 and 10 (Figure 1 **Panel B**) where the %TRL/remnant-C was highest and the causal association with inflammatory markers most evident. In models that included apoB and each of the biomarkers, the strength of association of apoB with risk was not diminished significantly by the inclusion of glycoprotein acetyls, CRP, white blood cell count, or blood neutrophil count (**Online Table 2**). Thus, using these statistical approaches there was no evidence that the causal relationship of TRL/remnants with systemic inflammation (as assessed by the four biomarkers) mediated the causal association of these lipoprotein particles with CHD risk.

### Genetically predicted variation in non-HDL-C and risk of CHD by SNP cluster

**Figure 3:**
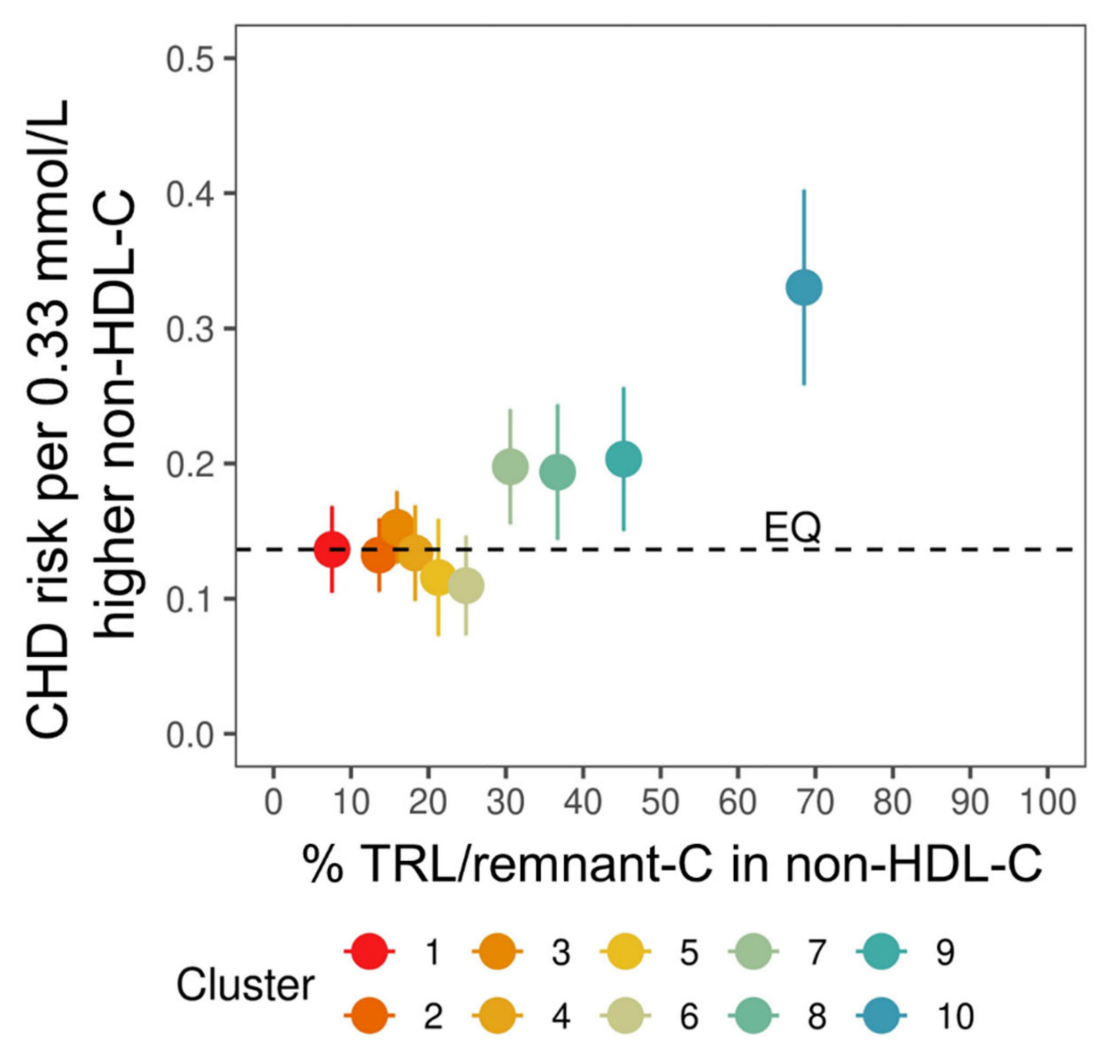
Non-HDL-cholesterol causal association with CHD risk. Genetically determined mean percentage TRL/remnant-C in non-HDL-C was derived for each SNP cluster as in Figure 1 **Panel B**. Mendelian randomisation analysis was performed to estimate for each SNP cluster the CHD odds ratio and standard error per 0.33 mmol/L increase in non-HDL-C. Theoretically, if the CHD risk per mmol/L increase in TRL/remnant-C is the same as the CHD risk per mmol/L increase in LDL-C then the odds ratio per 0.33 mmol/L of non-HDL-C should be constant across all 10 clusters (as denoted by the horizontal line labelled ‘EQ’) as the % TRL/remnant-C increase and the % LDL-C decreases.

Mendelian randomisation analyses were conducted to estimate the CHD risk per standardised increase (0.33 mmol/L) in non-HDL-C for each of the 10 SNP clusters (Table 2, Figure 3). Conceptually, if a given amount of cholesterol in TRL/remnants was associated with the same risk as in LDL then the CHD risk per mmol/L should be constant across the SNP clusters as depicted by the horizontal dashed line (‘EQ’) in Figure 3. However, this was not the case. CHD risk per 0.33 mmol/L increased in a graded manner as the genetically predicted percentage TRL/remnant-C in non-HDL-C rose and percentage LDL-C fell.

## Discussion

The principal finding from this study of the relationship of TRL/remnant particles to CHD risk is that plasma apoB and non-HDL-C do not exhibit a uniform quantitative causal association with CHD risk. As the abundance of TRL/remnants relative to LDL increases so does the risk per unit change in apoB, or per mmol/l of non-HDL-C. This is attributable to TRL/remnants having about a 4-fold higher per-particle atherogenicity than LDL. These results have pathogenic and clinical implications. Since non-HDL-C is used widely as a risk factor our observations indicate that the predicted risk requires adjusting upwards as the contribution of TRL/remnant-C to non-HDL-C rises. This is particularly the case in hypertriglyceridemia and in subjects on statin therapy where the TRL/remnant-C to LDL-C ratio is high.

Further, the observation that in Mendelian randomisation analysis inclusion of non-HDL-C rendered apoB a non-significant predictor of risk was noteworthy and suggested that the cholesterol content of particles rather than particle number (since plasma apoB reflects the total number of apoB-containing lipoproteins) was the better index of causal risk. However, the finding that for the same amount of non-HDL-C (0.33 mmol/L) risk increased as the percentage TRL/remnant-C increased indicated that properties of TRL/remnant particles other than a higher cholesterol content contributed to their atherogenicity. We found causal associations of TRL/remnants with circulating biomarkers of chronic systemin inflammation but were unable to show in mediation analysis that these relationships contributed independently to the causal association of TRL/remnants with CHD.

The results of this study can be set in the context of earlier publications^1,2,17^ that indicated TRL/remnant particles had potentially a greater – but until the present report yet to be quantified – per particle atherogenicity than LDL. Population studies based on Danish cohorts and our own analysis of the UK Biobank revealed that CHD risk per mmol/L of cholesterol was higher for TRL/remnant-C than for LDL-C.^2,17,18^ In our previous study we showed that a genetically predicted higher TRL-remnant/LDL ratio correlated with increased CHD risk per unit change in apoB.^2^ Similarly, Helgadottir et al^6^ found using a different SNP set that in both UK (a result we confirmed) and Icelandic data sets when non-HDL-C was introduced into a multivariable Mendelian randomisation model relating apoB to CHD risk, apoB became non-significant as a causal factor. These investigators attributed their results to inclusion of genetic variants that affected TG metabolism, a postulate which our analysis amplifies and explains. As seen in the present study, only when SNPs have an effect size on TRL/remnant-C that is sufficient to give a TRL/remnant-C to non-HDL-C ratio of >20% does the CHD risk (odds ratio) rise substantially above the value seen when LDL-C is the predominant non-HDL-C component.

A contrary view that all apoB-containing lipoproteins have an approximately equal quantitative association with CHD risk^3–5^ is based on evaluation of a smaller number of genetic instruments (SNPs linked to genes with known effects on lipid metabolism such as the LDL receptor or lipoprotein lipase genes). We believe in the context of lipoprotein physiology and the complex inter-relationships between lipoprotein species that the more ‘agnostic’ SNP cluster approach likely provides a more representative result, although the issue of pleiotropy has to be considered.^2,19–21^ Concerns regarding the interpretation of our genetic studies ^4^ have centred on the LDL-C measurement in the UK Biobank and comparison with discordancy studies. In the present investigation we used non-HDL-C as the key variable (obviating concerns regarding the LDL-C assay) and obtained results in accord with those previously published. Discordancy studies ask different questions of the data compared to Mendelian randomisation which determines average genetic associations between lipid variables and risk. The general agreement in investigations in distinct populations (UK Biobank, Icelandic, Danish and CARDIoGRAMplusC4D datasets), using different approaches is an indication of the robustness of the findings.

The finding that the degree of CHD risk causally associated with non-HDL-C varied depending on the percentage TRL/remnant-C implies that this widely used lipid biomarker will require adjustment to give a valid prediction of risk, either for all individuals, or more pragmatically when the percentage TRL/remnant-C in non-HDL-C exceeds 20%. This will be the case in individuals with raised TG such as those with type 2 diabetes,^1,8^ but also in a substantial proportion of the general population. In the UK Biobank the overall mean %TRL/remnant-C in non-HDL-C was 16%, and around 15% of subjects had ≥ 20% (associated with TG>2.5 mmol/L) while 25% of those on statin therapy had a %TRL/remnant-C in non-HDL-C > 20%.

The greater atherogenicity of TRL/remnants appeared not to be explained fully by their higher cholesterol content (the risk per unit cholesterol was higher in TRL/remnants than in LDL) and this raised the question as to what other features of these particles might have pathogenic actions such as pro-inflammatory effects.^22,23^ Population studies have demonstrated links between plasma TG, chronic inflammation, and CHD; TG levels are related strongly to CRP and to blood leucocyte count.^10,24^ In contrast, LDL-C is not associated with either variable. Our genetic analyses add to this picture in that we observed apparently causal associations between genetically predicted variation in TRL/remnants and both CHD risk and elevation in CRP, white blood cell count, blood neutrophil count and glycoprotein acetyls content. However, in mediation analyses we were not able to show statistically that the causal effect on these four biomarkers of systemic inflammation accounted for the causal association of TRL/remnants with CHD risk. This observation does not discount the possibility that a local or indirect inflammatory action of TRL on the artery wall promotes atherosclerosis in a way not reflected in systemic biomarkers.

The finding that TRL/remnants are considerably more atherogenic than LDL prompts the question as to how this observation aligns with the result of the PROMINENT trial^25^ where TG and very-low density lipoprotein cholesterol (VLDL-C, the measure of TRL/remnant-C used in the study) were reduced about 20% but no risk reduction was observed. The decrease in VLDL-C in the actively treated arm was 0.22 mmol/L, but this was accompanied by a 0.25 mmol/L increase in LDL-C and a 4 mg/dl rise in apoB. Extrapolating from the findings in the present investigation it can be predicted (as detailed in **Online Table 4**) that PROMINENT should have seen a relative risk reduction of approximately 6-7% in the pemafibrate arm over the 3.4-year trial duration. This predicted result falls within the confidence limits of the observed outcome - a hazard ratio of 1.03 with 95% CI of 0.91 to 1.15. Accordingly, our findings are not in disagreement with the trial results and emphasise the fact that an overall increase in apoB-containing particles can offset reduction in TRL/remnants even if the latter carry more risk per-particle.

### Study strengths and limitations

Our findings are based mainly on both total apoB and non-HDL-C measurements that were produced using established laboratory assays. The main results were replicated in a separate cohort (CARDIOGRAMplus C4D) and are in line with those seen in other populations. There are notable methodological limitations. TRL/remnant-C was calculated not measured. Separate VLDL (TRL/remnant)-apoB and LDL-apoB measurements were not available, and neither were apoB48 measurements that would have allowed a distinction to be made between chylomicrons and their remnants and VLDL. There are demographic limitations also since the UK Biobank comprises subjects of mainly White European ancestry. Finally, the SNP cluster approach is subject to potential confounding by pleiotropic actions.^2^

In conclusion, our results show that non-HDL-C and plasma apoB vary in their causal quantitative association with CHD, and that TRL/remnants are about four times more atherogenic than LDL on a per-particle basis. These findings prompt further investigation of the pathogenic features of TRL/remnant particles. They also highlight the potential basis on which clinical benefit from the reduction of TRL/remnants with diet or drug intervention might be seen. The implications of non-HDL-C having a variable quantitative relationship to CHD risk requires further investigation and confirmation since this is a widely used risk factor and treatment guide, especially in subjects with hypertriglyceridemia and in those on statin therapy.

## Clinical Perspectives

### Competency in Medical Knowledge

Hypercholesterolaemia is causatively linked to CHD and lowering of LDL reduces, but does not eliminate, risk of an atherosclerotic cardiovascular event, Recent advances in human genetics, together with epidemiologic and clinical trial results, indicate that the ‘residual’ ASCVD risk is in part caused by elevated levels of TRLs and their remnants. Here we show that TRL/remnant particles are about 4-fold more atherogenic than LDL on a per-particle (per apoB) basis, and that the causal relationship of non-HDL-C (per mmol/L) to CHD risk is not constant. These results have pathogenic and clinical implications. Since non-HDL-C is used widely as a risk factor in populations and for individuals, our observations indicate that the predicted risk may require adjusting upwards as the contribution of TRL/remnant-C to non-HDL-C rises.

### Translational Outlook

Studies in primary cells and animal models using TRL/remnants isolated from humans with hypertriglyceridemia are needed to elucidate the underlying mechanisms as to why TRL/remnants are more atherogenic than LDL particles.

## Supplementary Material

Supplementary material

## Figures and Tables

**Table 1:** Multivariable Mendelian randomisation models^a^ of apoB plus lipid variables and risk of an CHD event.

Multivariable MR models	CHD causal effect estimate (OR per unit change [95% CI])^b^	P-value
**Model 1**		
ApoB	1.10 (1.08–1.13)	8.6×10^-15^
TRL/remnant-C	1.41 (1.30–1.54)	5.5×10^-15^
**Model 2**		
ApoB	1.17 1.15–1.19)	9.4×10^-82^
TG	1.24 (1.18–1.32)	1.2×10^-14^
**Model 3**		
ApoB	1.00 (0.93–1.07)	0.93
Non-HDL-C	1.16 (1.10–1.23)	1.9×10^-7^
**Model 4**		
LDL-C	1.10 (1.07–1.13)	2.0×10^-13^
TRL/remnant-C	1.44 (1.32–1.57)	1.2×10^-16^

a Multivariable randomisation models used the 1357 SNP identified by GWAS and the inverse-variance weighted method for odds ratio (OR) calculation.

b Odds Ratio are expressed per 10 mg/dL increase in apoB, per 0.33 mmol/L (13 mg/dL) increase in LDL-C and TRL/remnant-C, and per 1 mmol/l increase in TG.

**Table 2:** Cluster specific CHD risk associated with genetically determined variation in non-HDL-C and apoB.

SNP set	No. of SNPs	Non-HDL-C composition % TRL-C % LDL-C		OR per 10 mg/dl higher apoB (95% CI)	OR per 0.33 mmol/L higher non-HDL-C (95% CI)*	P-value**
All SNPs	1357	25.5	74.5	1.20 (1.18–1.22)	1.16 (1.15–1.18)	3.0x10^-127^
Cluster 1	136	7.6	92.4	1.15 (1.11–1.19)	1.15 (1.11–1.19)	1.1×10^-15^
Cluster 2	136	13.6	86.4	1.17 (1.13–1.21)	1.14 (1.11–1.17)	5.6×10^-19^
Cluster 3	136	15.9	84.1	1.20 (1.16–1.24)	1.17 (1.14–1.2)	1.1×10^-28^
Cluster 4	136	18.3	81.7	1.18 (1.13–1.24)	1.14 (1.1–1.19)	4.0×10^-14^
Cluster 5	136	21.3	78.7	1.17 (1.11–1.24)	1.12 (1.08–1.17)	3.2×10^-08^
Cluster 6	136	24.9	75.1	1.17 (1.10–1.23)	1.12 (1.08–1.16)	2.1×10^-08^
Cluster 7	136	30.6	69.4	1.33 (1.25–1.41)	1.22 (1.17–1.27)	1.6×10^-20^
Cluster 8	135	36.7	63.3	1.34 (1.25–1.45)	1.22 (1.16–1.28)	5.6×10^-15^
Cluster 9	135	45.2	54.8	1.42 (1.31–1.54)	1.23 (1.16–1.30)	1.9×10^-17^
Cluster 10	135	68.5	31.5	1.70 (1.52–1.90)	1.40 (1.30–1.50)	3.5×10^-20^

* Odds ratios (OR) are expressed per 10 mg/dl increase in apoB and per 0.33 mmol/L (13 mg/dL) increase in non-HDL-C.

** P-values refer to the significance of the estimated odds ratios per 10 mg/dL apoB increase.

**Table 3:** Per-particle atherogenicity of TRL/remnants relative to LDL.

	Comparison	Estimated per-particle atherogenicity of TRL/remnants relative to LDL	95% CI
**UK Biobank**			
10 cluster analysis	Cluster 10 vs 1	3.9	2.8–5.4
20 cluster analysis	Cluster 20 vs 1	4.7	2.6–8.6
**Replication cohort**			
CARDIoGRAMplusC4D (outcome MI)	Cluster 10 vs 1	2.9	1.8–4.3

## Condensed Abstract

This SNP cluster-based study of the relationship of TRL/remnant particles to coronary heart disease risk revealed that TRL/remnants were about 4 times more atherogenic than LDL on a per-particle (per apoB) basis. The causal relationship of non-HDL-C to CHD risk (per mmol/L) is not constant. Rather, greater genetically predicted TRL/remnant-C within non-HDL-C led to increased risk, underscoring the higher atherogenicity of TRL/remnants. These findings have significant pathogenic and clinical implications. Given the widespread use of non-HDL-C as a risk indicator, our observations suggest the need for upward adjustments in predicted risk as TRL/remnant-C contribution to non-HDL-C increases.

## Central Illustration

**TRLs and their remnants are markedly more atherogenic than LDL**

Genetic variants were categorized based on their impact on TRL/remnant-C versus LDL-C and grouped into 10 clusters. Cluster 1 SNPs predominantly affected LDL and cluster 10 predominantly TRL/remnants.

If all apoB-containing lipoprotein particles were equally atherogenic, then the risk per 10 mg/dL increase in apoB should be consistent and the CHD risk per unit apoB should be horizontal. However, this wasn’t observed; risk per apoB increased as the relative abundance of TRL/remnants rose. TRL/remnants were about four times more atherogenic per particle compared to LDL.

Likewise, if TRL/remnant-C and LDL-C were equally atherogenic then the CHD risk per unit non-HDL-C should have given a horizontal line as TRL/remnant-C replaced LDL-C (mmol for mmol). The observation of positive gradient as TRL/remnant-C increased and LDL-C decreased indicates that cholesterol in TRL/remnant particles carries a higher risk than cholesterol in LDL particles: a finding with implications for risk prediction using non-HDL-C.
