# Supplementary material for: Quantifying triglyceride-rich lipoprotein atherogenicity, associations with inflammation, and implications for risk assessment using non-HDL cholesterol
Source: J Am Coll Cardiol. Author manuscript; Available in PMC 2024 Oct 31. (PMC7616757; doi:10.1016/j.jacc.2024.07.034)
Supplement: Supplementary material [file EMS198948-supplement-Supplementary_material.pdf]

SUPPLEMENTAL ONLINE MATERIAL

Quantifying triglyceride-rich lipoprotein atherogenicity, associations with inflammation, and implications for risk assessment using non-HDL cholesterol.

Content

Page	Element included
2	Online Table 1. Numbers and definition of CHD outcomes
3	Online Table 2. Potential mediating effect of inflammatory markers on the relationship between apoB (predominantly in TRL/remnants) and CHD
4	Online Table 3. Mendelian Randomization results using the MR Median, MR Conmix and MR Egger methods.
5	Online Table 4. Prediction of the PROMINENT trial outcome
6	Online Figure 1. Flowchart of subject- and SNP selection as basis for the Mendelian Randomisation analyses
7	Online Figure 2. Rationale for calculation of TRL/remnant per-particle atherogenicity relative to LDL
8	Online Figure 3. Relationships between genetically predicted variation in TRL/remnant-C, TG and LDL-C in defined clusters of SNPs from GWAS SNP set
9	Online Figure 4. Replication of main result in the CARDIoGRAMplusC4D cohort
10	Online Figure 5. Sensitivity analyses: (1) using MI only as outcome and (2) excluding apoE2 homozygotes.
11	References

ON-LINE TABLE 1. Numbers and definition of CHD outcomes

**CHD outcome**		**Individuals, n=487,202**
Non-fatal myocardial infarction (MI)	ICD 9 codes 410, 4110, 412, 42979<br>ICD 10 codes I21, I22, I23, I241, I252	Prevalent events n = 6,577<br>Incident events n = 17,356
Fatal MI	ICD 10 codes I21, I23, I241, I251, 1252, I253, I255-I259	Incident events n = 3,850
Coronary revascularisation	**Operational procedures**<br>Codes K501, K40-K44	Prevalent events n = 2,845<br>Incident events n = 3,571
Unique CHD outcomes	First event of above	Prevalent events n = 8,391<br>Incident events n = 20,792<br>Total events = 29,183

Incident events based on approximately 13 years of follow (as of Jan 2021).

ONLINE TABLE 2. Potential mediating effect of inflammatory markers on the relationship between apoB (predominantly in TRL/remnants) and CHD

	Cluster 10		Cluster 9	
	Estimate (95% CI)	P-value	Estimate (95% CI)	P-value
**Mediation analysis: ApoB -> CRP -> CHD**				
Total effect	5.92 (5.1–6.8)	<0.001	3.52 (2.6–4.5)	<0.001
ApoB-effect	5.97 (5.1–6.9)	<0.001	3.04 (2.0–4.0)	<0.001
CRP-effect	-0.05 (-0.2–0.1)	0.462	0.48 (0.0–1.0)	0.074
Proportion mediated by CRP (%)	-0.6	0.462	13.7	0.074
**Mediation analysis: ApoB -> GPA -> CHD**				
Total effect	5.96 (5.0–6.9)	<0.001	3.49 (2.6–4.4)	<0.001
ApoB-effect	5.4 (3.9–6.9)	<0.001	4.39 (2.2–6.4)	<0.001
GPA-effect	0.56 (-0.7–1.8)	0.374	-0.9 (-2.9–1.1)	0.36
Proportion mediated by GPA (%)	9.4	0.374	-26.2	0.36
**Mediation analysis: ApoB -> WBC -> CHD**				
Total effect	5.88 (5.0–6.9)	<0.001	3.51 (2.6–4.4)	<0.001
ApoB-effect	5.84 (4.9–6.9)	<0.001	3.16 (2.2–4.2)	<0.001
WBC-effect	0.04 (-0.3–0.4)	0.786	0.36 (0.0–0.8)	0.078
Proportion mediated by WBC (%)	0.6	0.786	9.7	0.078
**Mediation analysis: ApoB -> Neutrophils -> CHD**				
Total effect	5.89 (5.0–6.8)	<0.001	3.51 (2.6–4.4)	<0.001
ApoB-effect	5.89 (4.9–6.9)	<0.001	3.0 (2.0–3.9)	<0.001
Neutrophil-effect	0.025 (-0.42–0.41)	0.964	0.53 (0.15–0.98)	0.004
Proportion mediated by WBC (%)	0.1	0.964	15	0.004
**Univariable MR model**				
ApoB term	5.31 (4.2–6.4)	<0.001	3.52 (2.7–4.3)	<0.001
**Multivariable MR model (adjusted for inflammatory markers)**				
ApoB term	3.81 (1.8–5.8)	<0.001	3.70 (1.9–5.5)	<0.001
GPA term	2.34 (-0.12–4.8)	0.062	-1.50 (-4.9–1.9)	0.38
CRP term	-0.06 (-0.2–0.07)	0.36	0.08 (-0.07–0.23)	0.29
WBC term	-0.11 (-0.59–0.34)	0.61	-0.05 (-0.54–0.44)	0.83
Neutrophil term	0.14 (-0.58–0.88)	0.68	0.34 (-0.44–1.1)	0.39

Potential mediating role of the inflammatory markers C-reactive protein (CRP), glycoprotein acetyls (GPA), white blood cell count (WBC) and neutrophil count was investigated using mediation analysis and multivariable MR analysis for the two clusters showing the largest effect on TRL/remnants (cluster 9 and 10, main text **Figure 2**). Mediation analysis tests whether the total effect on CHD outcome is a result of a direct effect of the apoB containing lipoprotein or it is mediated by CRP, GPA, WBC or neutrophil count. We find no evidence of mediating effects of these inflammatory biomarkers. Additionally, when adjusting for the inflammatory biomarkers in a multivariable MR model, we find that apoB generally retains its robust effect on CHD and that the inflammatory biomarkers are not significantly associated with CHD alone.

ONLINE TABLE 3. Mendelian Randomization results using the MR Median, MR Conmix and MR Egger methods. Estimates are odds ratios per 10 mg/dL apoB. Results are broadly similar to the inverse-variance weighted method (main text **Table 2**).

	MR Median estimate	MR Median p-value	MR Conmix estimate	MR Conmix p-value	MR Egger estimate	MR Egger p-value	MR Egger Intercept	MR Egger intercept p-value
Cluster 1	1.15	4.63E-13	1.15	4.81E-12	1.13	6.20E-08	0.00247	0.245
Cluster 2	1.13	1.12E-06	1.27	1.97E-12	1.1	6.51E-05	0.00625	0.0024
Cluster 3	1.16	2.53E-11	1.25	1.57E-13	1.19	1.49E-13	0.00196	0.385
Cluster 4	1.19	1.18E-06	1.27	4.29E-11	1.19	7.06E-06	-0.000106	0.965
Cluster 5	1.18	1.49E-06	1.18	3.44E-12	1.17	1.11E-03	0.000318	0.905
Cluster 6	1.14	2.90E-04	1.15	1.15E-21	1.14	4.51E-03	0.00168	0.492
Cluster 7	1.23	4.92E-08	1.3	2.46E-15	1.25	2.40E-04	0.00332	0.229
Cluster 8	1.35	3.57E-10	1.43	8.01E-10	1.25	6.48E-03	0.00316	0.28
Cluster 9	1.33	4.42E-07	1.56	1.95E-12	1.36	2.07E-06	0.00187	0.376
Cluster 10	1.71	6.36E-12	1.74	1.72E-26	1.69	6.30E-11	0.00023	0.901

ONLINE TABLE 4. Prediction of the PROMINENT trial outcome

Variables	Values
Lifetime log OR per 1 mmol/l TRL/remnant-C	1.10
Lifetime log OR per 1 mmol/l LDL-C	0.28
LDL-C change observed in PROMINENT (mmol/l)	+0.25
TRL-C/remnant-C change observed in PROMINENT (mmol/l)	-0.22
Predicted life-time log odds ratio	0.28*0.25 – 1.10*0.22 = -0.17
Predicted life-time odds ratio	0.84
Predicted life-time risk reduction (%)	16
Predicted risk reduction during 3.4-year trial* (%)	6.4

*Calculated as 40% of the life-time risk reduction estimated from Figure 4 in Wang et al.[1]

ONLINE FIGURE 1. Flowchart of subject- and SNP selection as basis for the Mendelian Randomisation analyses

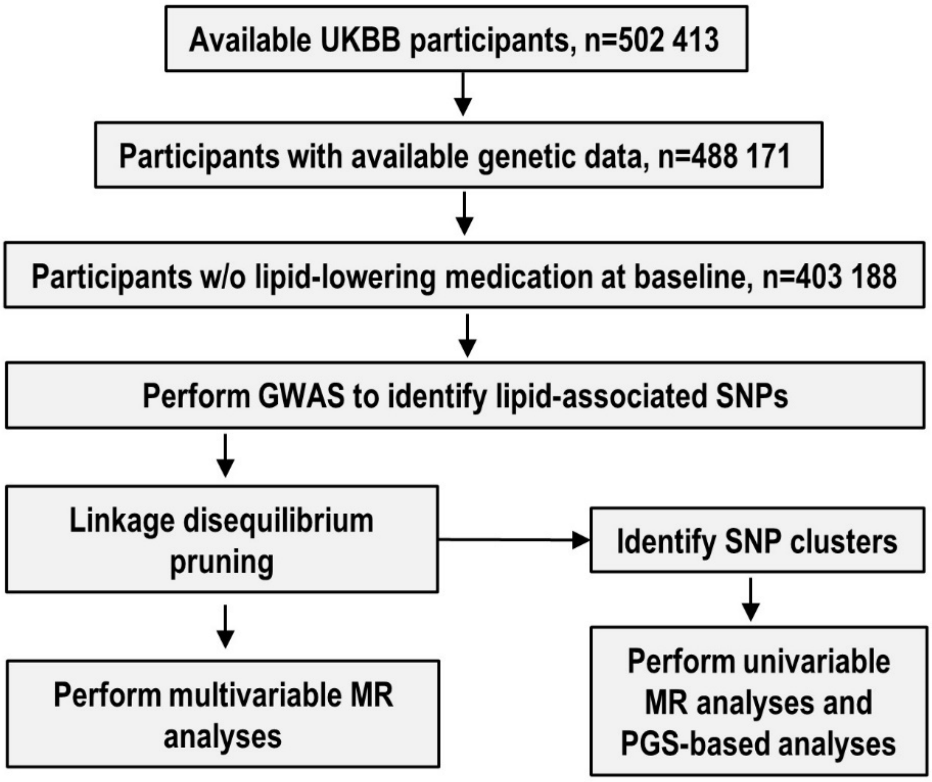

1. Genetic instruments (SNPs) and β-coefficients were determined in subjects not on lipid-lowering therapy in whom appropriate lipoprotein levels were available (in the case of TRL/remnant-C the number of subjects was 350,110). This selection criterion eliminated the confounding effects of drugs (mainly statins) on the SNP to exposure relationship.
2. The association of genetically-determined variation in lipoprotein variables with CHD outcome was explored using all subjects with appropriate genetic information available. The number of subjects in these analyses was 487,202 and included those off or on lipid-lowering treatment.
3. Analysis of the relationship between observed lipoprotein variables measured at baseline and incident CHD was undertaken in subjects free of CHD at baseline. The number of subjects meeting this criterion was 478,811.

ONLINE FIGURE 2. Rationale for calculation of TRL/remnant per-particle atherogenicity relative to LDL

Our approach to determining a per-particle atherogenicity is based on the following:

a) each TRL/remnant and LDL particle contain only one apoB protein. Thus, any change in apoB concentration reflects a change in particle number.

b) Mendelian randomisation analyses provided an estimate of the average effect (beta-coefficient) of an allelic variant (SNP) on plasma apoB and the associated effect on CHD risk (odds ratio). We used 10mg/dL as a standardised change to compare SNP cluster effects.

c) For each SNP cluster the genetically determined change in plasma apoB will be the sum of the changes in TRL/remnant-apoB and LDL-apoB.

% TRL/remnant-C in non-HDL-C as an index of %TRL-apoB in plasma apoB.

The UK Biobank data set provides plasma total apoB but not apoB levels for TRL/remnants (VLDL) and LDL separately. However, we can in apportion the genetically determined increase in plasma apoB to TRL/remnants or to LDL depending on the effect of the cluster-specific SNPs on *%TRL/remnant-C in non-HDL-C* which in theory can range from 0% (SNPs had no effect on TRL/remnants) to 100% (SNPs have no effect on LDL).

- **LDL-apoB:** If the SNPs in a cluster have an average effect on TRL/remnant-C that is at or close to 0%, then the genetically determined change in plasma apoB (standardised to 10mg/dL) can be assigned to a change in LDL-apoB. This is the case for SNP cluster 1 in the ventile analysis (**main text Figure 1C**) where the SNP effects on %TRL/remnant-C were <5%. The decile analysis (**Table 1**) gave a similar result.

- **TRL/remnant-apoB:** SNP clusters with the greatest effect on TRL/remnants did not produce a %TRL/remnant-C close to 100%. However, in the ventile analysis SNP cluster 20 gave a value of ~80% (main text **Figure 1C**). Using literature values for the cholesterol to apoB ratio in TRL/remnants of 1.5 and in LDL of 1.0,[2] it can be calculated that the change in TRL/remnant-apoB as a % of plasma total apoB was 73%. Thus, about three quarters of the 10mg/dl change in plasma apoB can be assigned to TRL/remnant-apoB.

An alternate approach is to extrapolate a regression line through the experimentally derived CHD odds ratios for clusters 1 to 20 to 100%TRL/remnant-C. This then as noted above allows us to assign the 10mg/dL change in plasma apoB entirely to TRL/remnant-apoB.

In conclusion

We can with reasonable confidence determine for the lowest SNP cluster in the decile or ventile analysis a CHD odds ratio per 10mg/dL change in LDL-apoB. For the topmost SNP cluster (cluster 10 of the decile, cluster 20 of the ventile analysis) we can provide a conservative/ minimum estimate of the CHD odds ratio per 10 mg/dL of TRL/remnant-apoB. Also, by extrapolation we can generate an estimated odds ratio (especially in the ventile analysis) that is associated entirely with a change in TRL/remnant-apoB.

Dividing the log odds ratio for TRL/remnant-apoB by the log odds ratio for LDL-apoB gives an estimate for the relative atherogenicity of the particles.

ONLINE FIGURE 3. Relationships between genetically predicted variation in TRL/remnant-C, TG and LDL-C in defined clusters of SNPs from GWAS SNP set.

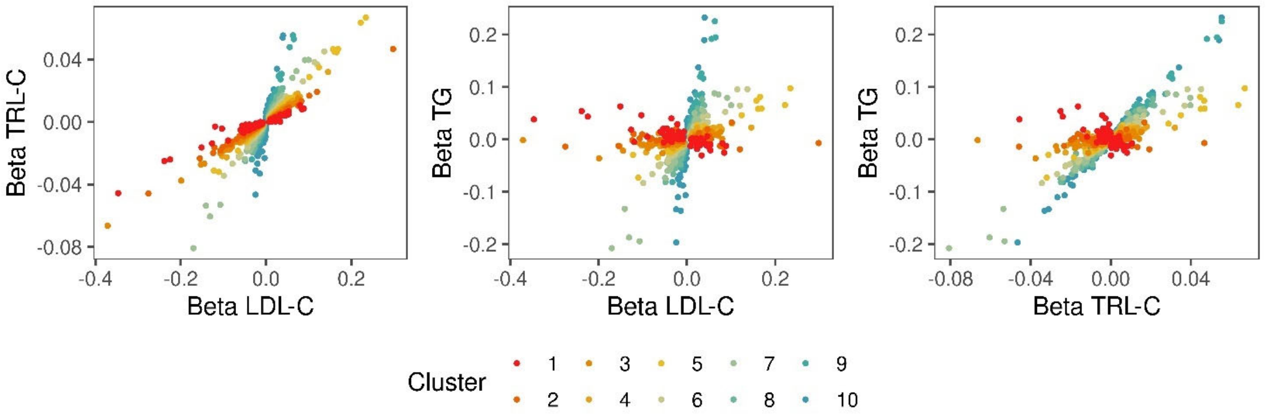

The GWAS SNP set was allocated to clusters 1 to 10 based on the ratio of β-coefficients for TRL/remnant-C and LDL-C (**Figure 1**). The panels above show the association of TRL/remnant-C and TG to LDL-C for clusters 1-10. Units are mmol/l for each variable.

For cluster 1 genetically predicted variation in LDL-C was accompanied by a range of effect sizes for TRL/remnant-C (**Panel A**) but there was only a weak association, if any, with TG (**Panel B**). Cluster 10 SNPs, in contrast, had relatively large effects on TG but only small effects on LDL-C (**Panel B**). Comparing the effect sizes of TRL/remnant-C and TG (**Panel C**), it was observed that while SNPs in clusters 1 to 10 had approximately the same range of effect sizes for TRL/remnant-C, the associated effect sizes for TG varied within a narrow interval for clusters 1-3 and a much broader interval for clusters 8-10.

ONLINE FIGURE 4. Replication of main result in the CARDIoGRAMplusC4D cohort

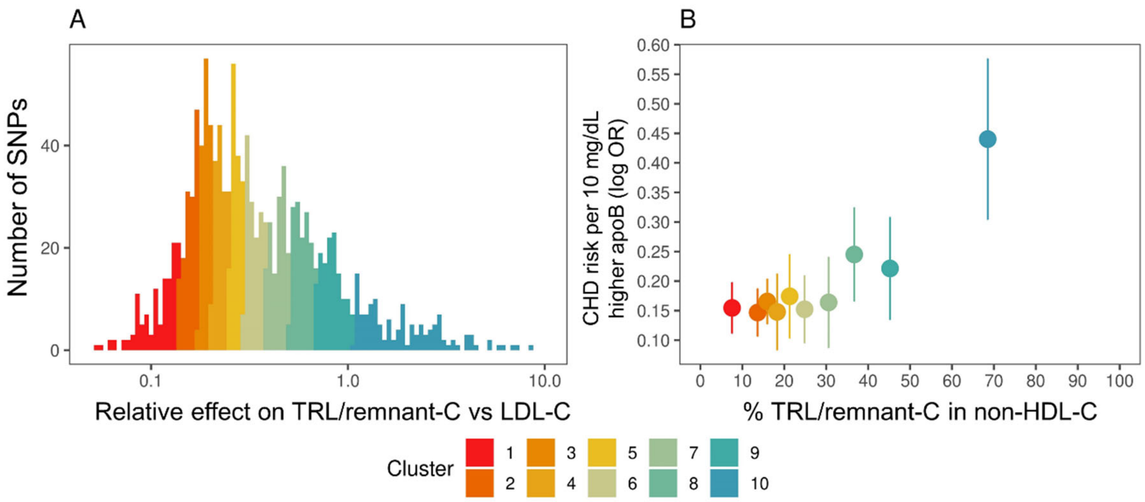

SNP β-coefficients for TRL/remnant-C and LDL-C derived from the UK Biobank were applied to the CARDIoGRAMplusC4D data set to generate odds ratios for each SNP cluster. Panel B show odds ratios per 10 mg/dl apoB versus genetically determined %TRL/remnant-C in non-HDL-C.

ONLINE FIGURE 5. Sensitivity analyses: (1) using MI only as outcome and (2) excluding apoE2 homozygotes.

1. **Choice of CHD outcome.**
   The outcome measure we employed in all analyses was the combination of fatal/ non-fatal MI plus revascularisation. To ascertain if this choice affected the relationship seen between %TRL/remnant-C in non-HDL-C and CHD risk per unit change in apoB, a further analysis was performed using MI only as the outcome. The results shown in the second panel below were virtually identical with the original (combination outcome) data as seen shown in the first panel.
   The number of revascularisations was about one-fifth that of MI and this was considered not to offer sufficient statistical power to be used as an outcome by itself.

2. **Influence of dysbetalipoproteinaemia/type III hyperlipidaemia.**
   The possibility that the relationship between %TRL/remnant-C in non-HDL-C and CHD risk per unit change in apoB was overly influenced by dysbetalipoproteinaemia was explored. The theoretical concern is that this dyslipidaemia which is uncommon but has a profound effect on the plasma lipid profile might have distorted the association seen in the whole cohort to give the pattern we observed in **Figure 1 Panel B** (shown also in the first panel below). Dysbetalipoproteinaemia occurs when subjects are homozygous for a defect in the apoE gene giving rise to the apoE2 variant protein that cannot bind to lipoprotein receptors. If there is no lipid-raising factor present, then this apoE2 homozygosity causes remnant lipoproteins to accumulate in the circulation and there is a reduced LDL-C level but no overall increase in apoB or non-HDL-C. If there are lifestyle or genetic factors that increase lipid levels, then frank Type III hyperlipidaemia develops.
   Fortunately, a specific SNP is available to test for the variant of the *APOE* gene that codes for apoE2. We repeated our central analysis excluding homozygotes for this SNP (rs7412) and observed as seen in the third panel below that the exclusion of apoE2/E2 subjects had no effect on the observed relationship. We concluded that the overall associations we found were not explained by the influence of this uncommon genotype/phenotype.

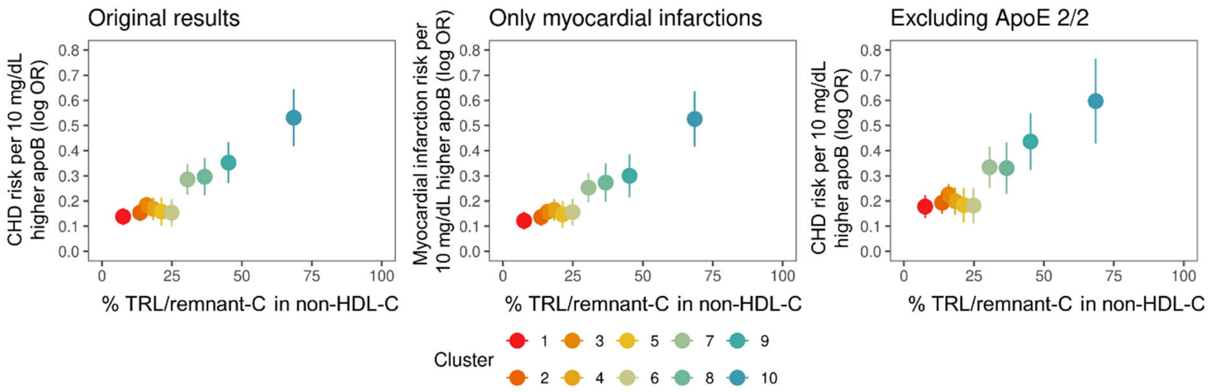

REFERENCES

1. Wang N, Woodward M, Huffman MD, Rodgers A. Compounding Benefits of Cholesterol-Lowering Therapy for the Reduction of Major Cardiovascular Events: Systematic Review and Meta-Analysis. *Circ Cardiovasc Qual Outcomes*. 2022;15:e008552.
2. Zambon A, Deeb SS, Bensadoun A, Foster KE, Brunzell JD. In vivo evidence of a role for hepatic lipase in human apoB-containing lipoprotein metabolism, independent of its lipolytic activity. *J Lipid Res*. 2000;41:2094-2099.
